# Nitrogen balance and supply in Australasian mushroom composts

**DOI:** 10.1007/s00253-023-12933-2

**Published:** 2024-01-19

**Authors:** Ralph Noble, Meghann Thai, Michael A. Kertesz

**Affiliations:** 1 Microbiotech Ltd, Pershore Centre, Pershore, Worcestershire WR103JP UK; 2 Faculty of Science, School of Life and Environmental Sciences, The University of Sydney, LEES Building, Sydney, NSW 2006 Australia

**Keywords:** Nitrogen, Mushrooms, *Agaricus bisporus*, Ammonia, Nutrient cycling, Circular economy

## Abstract

**Abstract:**

Mushrooms are an important source of protein in the human diet. They are increasingly viewed as a sustainable meat replacement in an era of growing populations, with button mushrooms (*Agaricus bisporus)* the most popular and economically important mushroom in Europe, Australia and North America. Button mushrooms are cultivated on a defined, straw-derived compost, and the nitrogen (N) required to grow these high-protein foods is provided mainly by the addition of poultry manure and horse manure. Using the correct balance of carbon (C) and N sources to produce mushroom compost is critically important in achieving maximum mushroom yields. Changes in the amount and form of N added, the rate and timing of N addition and the other compost components used can dramatically change the proportion of added N recovered in the mushroom caps, the yield and quality of the mushrooms and the loss of N as ammonia and nitrogen oxide gases during composting. This review examines how N supply for mushroom production can be optimised by the use of a broad range of inorganic and organic N sources for mushroom composting, together with the use of recycled compost leachate, gypsum and protein-rich supplements. Integrating this knowledge into our current molecular understanding of mushroom compost biology will provide a pathway for the development of sustainable solutions in mushroom production that will contribute strongly to the circular economy.

**Key points:**

• *Nitrogen for production of mushroom compost can be provided as a much wider range of organic feedstocks or inorganic compounds than currently used*

• *Most of the nitrogen used in production of mushroom compost is not recovered as protein in the mushroom crop*

• *The sustainability of mushroom cropping would be increased through alternative nitrogen management during composting and cropping*

## Introduction

Mushrooms are an increasingly important source of nutrition worldwide, and annual mushroom production has grown over 30-fold in the last 40 years (Royse et al. 2017). Although oyster mushrooms (*Pleurotus*) and shiitake (*Lentinula*) dominate this production globally, in Europe, the USA and Australia the button mushroom (*Agaricus bisporus*) is commercially the most important cultivated mushroom. Button mushrooms are grown on a composted substrate derived mainly from wheat straw, stable bedding (horse manure), poultry manure and gypsum. This varies regionally, however, with horse manure playing a large role in Europe and the USA, and rice straw commonly replacing wheat straw in China (Song et al. 2021). Smaller amounts of other materials are used to provide bulk or nitrogen (N) input, depending on seasonal availability, and although mushroom compost is a more defined substrate than green-waste composts (which may contain woody materials, grasses and leaves at certain seasons), materials such as canola, soybean, cottonseed and sugarcane bagasse are often added to stimulate microbial activity. The composting process varies somewhat between countries, but usually includes an initial period of wetting or soaking of the raw materials, a thermophilic composting period (Phase I, 70–80 °C) and a pasteurisation and conditioning step (Phase II, 60 °C; then decreasing to 45 °C).

**Fig. 1:**
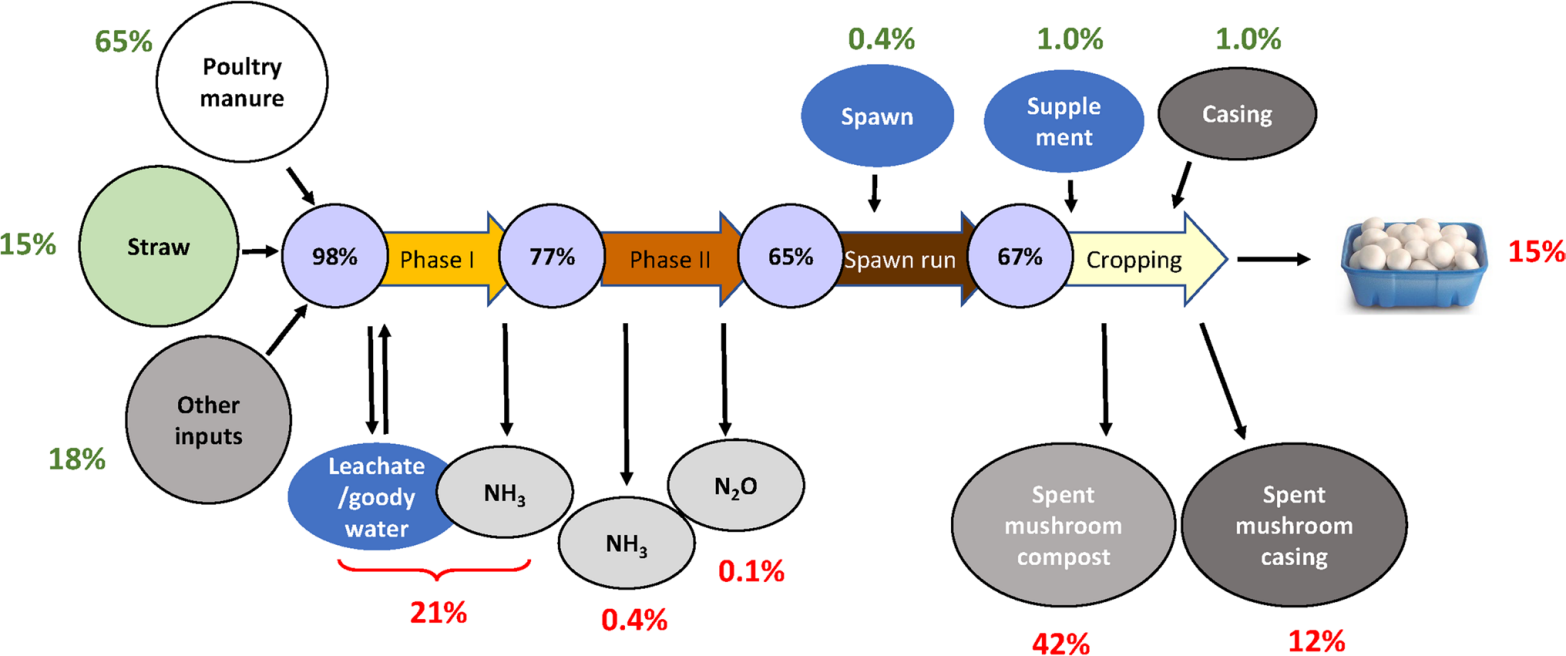
Nitrogen balance in button mushroom production. Values are shown as percentage of total input nitrogen. Green numbers, N inputs; red numbers, N outputs; purple numbers, derived from compost N content measurements, allowing for volume losses during composting. The data are average values from ten Australian mushroom composting facilities. Losses during Phase 1 are estimated, since NH_3_ losses could not be measured and the proportion of recycled leachate used in composting varied greatly between facilities. Data from Thai (2022).

The composting process relies on microbial activity to break down the lignocellulosic raw materials and incorporate added N into microbial biomass in the compost. The microbial community in the compost changes continuously during composting, responding to changes in temperature and the progressive assimilation of plant cell components, starting with readily available compounds such as lipids and sugars, and progressing to polymers such as cellulose, hemicelluloses and lignin. The microbial dynamics of this process have been studied in detail in recent years, focussing largely on phylogenetic profiling (Cao et al. 2019; Carrasco et al. 2020; Song et al. 2021; Thai et al. 2022; Vieira and Pecchia 2021). Investigations of changes in the functional diversity in mushroom compost have concentrated primarily on enzymes responsible for lignocellulose breakdown to provide carbon for growth (Chang et al. 2022; Kabel et al. 2017; Zhang et al. 2014). Lignocellulose has only a very low N content, so the polysaccharide-degrading bacteria rely on particular strategies for assimilation of available nitrogen while degrading the straw polysaccharides (Gardner and Schreier 2021). For compost in particular, the key nitrogen-transforming activities observed are high levels of proteolysis and ammonification in Phase I and high levels of nitrification during Phase II conditioning (Caceres et al. 2018). This leads to significant losses of nitrogen as ammonia in the thermophilic phase of composting, and conversely, reassimilation of ammonia during conditioning. In Australasian composting facilities, about 30% of the added nitrogen is lost during Phase I and a further 10% during Phase II (Fig. 1) (Thai 2022). Because of these losses, it is usual to add a nitrogenous supplement to the compost before cropping, but this constitutes only a small proportion of the total nitrogen already present in the compost. Within our diet, mushrooms are a source of essential fatty acids (Sande et al. 2019) and major and minor trace elements (Siwulski et al. 2020) and are also regarded as a high protein food (Wang and Zhao 2023). However, only about 15% of the total added nitrogen to the composting process is recovered in the mushroom crop. This highlights that most of the nitrogen that is added in the composting process is not actually required for mushroom fructification, but is important in promoting the composting process itself. Nitrogen is required to stimulate the microbial activity that is important in creating a productive compost, but in the form of ammonia, it is also very important in preparing the compost substrate chemically for enzymatic degradation. Proteolysis generates significant quantities of ammonia during Phase I composting, and at the elevated pH and temperature conditions present this ammonia helps promote chemical degradation of hemicellulose and lignin (Mouthier et al. 2017). This principle applies not only to mushroom composts—the process of general organic waste composting also depends crucially on transformation of individual N fractions in the feedstocks (Estrella-Gonzalez et al. 2020).

Australian and New Zealand mushroom farms grow around 60,000 tonnes of button mushrooms (*Agaricus bisporus*) annually (Food and agriculture organisation 2019). This requires around 340,000 tonnes of Phase I compost, which is manufactured from about 260,000 tonnes of raw materials (den Ouden 2016; Gerrits 1988). Australasian mushroom compost is almost entirely produced from a wheat straw/poultry manure/gypsum blend, with minimal additions of other ingredients such as horse manure and occasional supplementation with small amounts of N in forms such as urea or ammonium sulphate. The feedstock composition used is very consistent through the year, and in recent years, most Australasian mushroom compost facilities have moved to large, forced-aeration bunker systems with uniformly high temperatures and short composting times, replacing the slow traditional methods of turned windrows. This consistency of feedstocks and process makes Australasian mushroom compost a useful subject for detailed studies of the mushroom composting process, but it also means that the Australasian mushroom industry is very dependent on the availability of a small range of raw materials in order to maintain economic viability.

The 1990s and early 2000s were a ‘golden age’ of empirical research into composting and particularly the production of *Agaricus*-selective mushroom compost. Extensive studies were done to choose optimal feedstocks of C and N for composting, and to predict crop yields from the inputs and from the physicochemical parameters of compost. Progress has been slower since then, but with the advent of high throughput molecular tools (especially sequencing technologies), there has been a renaissance in studies of compost microbial diversity and development and its impact on mushroom yield and quality. Several reviews have appeared that summarise aspects of mushroom cropping and the biology of *Agaricus bisporus* (Baars et al. 2020; Carrasco et al. 2018; McGee 2018) and review bacterial-fungal interactions in mushroom compost (Braat et al. 2022; Carrasco and Preston 2020; Kertesz and Thai 2018; Shamugami and Kertesz 2023) and mushroom production as part of the circular economy (Grimm and Wosten 2018).

This current report addresses a different critical aspect of composting, and directs the reader’s attention back to questions of how best to manage microbial nutrient management in the production of mushroom compost. It aims to summarise and synthesise earlier findings so that modern researchers are able to integrate previous conclusions (including insights from less accessible industry and conference sources) into recent advances in our understanding of mushroom compost biology. This will allow researchers to avoid duplication of previous research and provide a pathway for the pursuit of productive new directions and the development of sustainable solutions that will contribute more strongly to the circular economy.

## Carbon and nitrogen sources in mushroom compost

Achieving the correct balance of carbon (C) and N sources in mushroom compost is important in achieving maximum mushroom yields. During composting, N is used by the compost microbiota to degrade some of the cellulose and hemicellulose in straw, the main C source in compost, into microbial biomass and high molecular weight polymers, making it a more selective nutrient source for the mushroom and less available for competitor micro-organisms (Fermor et al. 1985; Wood and Fermor 1985). Compost formulations deficient in N are therefore less productive overall than formulations in which there is an adequate supply of N (Gerrits 1988; Noble and Gaze 1994; O’Donoghue 1965). If adequate N is present when mushroom inoculum or ‘spawn’ is added to pasteurised (Phase 2) compost, mushroom yield does not change with a further slight increase in compost N contents, e.g. between about 2.1 and 2.7% of dry matter (DM) ((Cormican and Staunton 1991); Noble unpublished data). However, over-supply of N in compost formulations results in excessive evolution of ammonia and nitrous oxides and N losses (Noble et al. 2002). Some loss in N during composting by ammonification is almost inevitable and provides an available N-source for compost microbes, but ammonia is also toxic to the *Agaricus* mycelium, and composts with a high ammonium-N content, generally above 0.15% of DM at spawning, are less productive ((Cormican and Staunton 1991); Noble unpublished data). This limits the amount of N that can be added into compost formulations for conversion into microbial biomass. To increase mushroom yields, additional proteins are therefore usually added to the prepared composted substrate (Gerrits 1988), most commonly in the form of soya-based supplements (see later section on compost supplements).

The total C:N ratio is widely used by composters to determine optimum mushroom compost formulations, but this is an over-simplification, since it is the available C and N to micro-organisms that are important. However, for most compost ingredients, the total C and N are likely to give an indication of the available C and N (Gerrits 1977b). The optimum C:N ratio for the blended ingredients in a mushroom compost formulation is about 30:1, equivalent to an N content (including ammonium-N) of about 1.5% of DM (den Ouden 2016; Gerrits 1977b). At this level, the N losses as ammonia during the first stage or Phase I of composting are almost counterbalanced by the losses in C as carbon dioxide, so that the compost N content increases only slightly. At starting levels of N above 2%, more N is lost as ammonia than C as carbon dioxide, resulting in a decrease in compost N content during composting. When the starting level of compost N is below 1.5%, ammonia losses are small and the compost N content increases during composting (Gerrits 1977b; Gerrits 1988). Materials with N contents of more than 2% of DM can therefore be regarded primarily as N sources. Organic matter ingredients with N contents of less than 1% of DM can be regarded primarily as C sources, and those with intermediate N contents, such as horse manure, can also be regarded as significant or even sole sources of N.

## Straw as a source of C and N

The main component and C source in mushroom composts in temperate regions is wheat straw, used fresh or as horse manure, which may also contain proportions of other types of straw such as barley. The amount of C in wheat straw that is available to microbes varies widely between different straw sources. A study of 84 wheat straw samples from across the UK found that soluble carbohydrates varied between 3 and 19% and hemicellulose between 10 and 29%, even though total C content only ranged from 36 to 39% (Noble et al. 2006).

Wheat straw contains between 0.3 and 1.09% of N (Atkins 1974; Noble 2006) but it is unclear how much of this N can be used by microbes during composting. For example, rape straw containing 1.2% N produced a similar amount of ammonia during composting to wheat straw containing 0.5% N when the same amounts of poultry manure were added to each (Noble et al. 2002). This indicates that much of the N in straw is unavailable to microbes during composting.

Rye straw degrades and performs similarly to wheat straw for production of mushroom compost (Gerrits 1988; Noble and Dobrovin-Pennington 2007) and in Asia, rice straw is commonly used in place of wheat straw (Kim 1978; Noble et al. 2001). Oat and barley straw degrade more rapidly during composting than wheat straw (Gerrits 1988); this may necessitate shorter composting to avoid loss in structure and aeration. Noble and Gaze (1994) obtained significantly poorer mushroom yields from ‘environmentally controlled’ composts prepared from barley straw than from wheat straw although subsequent experiments with compost prepared in bunkers from barley straw yielded comparable yields to those from wheat straw (Noble et al. 2002). It is possible that the greater digestibility and availability of C in barley straw than in wheat straw may influence the optimum amounts and types of N that can be used for preparing mushroom substrates. Straw from sugarcane, rape, linseed, peas and beans, various grasses and corn cobs have also been used as C sources in mushroom compost formulations although complete replacement of wheat or similar straw has usually resulted in reduced mushroom yields (Noble et al. 1998; Poppe 2000).

High energy C sources such as molasses have also been included in compost formulations (Hayes et al. 1969), in order to increase the availability of C to compost microbiota and promote the rate and temperature of mushroom composting. Success with these additives led to the production of commercial compost ‘activators’ such as ADCO Sporavite (Noble et al. 1998). However, the addition of sugars to compost formulations did not shorten the time needed to clear ammonia from the compost or increase mushroom yields (Gerrits 1988). High compost temperatures, rapidly achieved in modern insulated and aerated bunker systems, have also made the use of such compost activators unnecessary.

## Organic matter N sources

Extensive lists of raw materials that can be used as substrates for mushroom cultivation were collated by Stamets (2000) and Poppe (2000), but many of these materials are only available in tropical regions and are less appropriate in temperate zones. N sources that may be available in quantity in Australasia include cow, pig and sheep manures; animal skin, hair, bone, dried blood and horn wastes; feathermeal, fish and shellfish residues; brewery and distillery wastes; and grape, citrus and olive fruit wastes. Although Australia and New Zealand produce huge quantities of animal manures, much of this material is widely dispersed and remote from mushroom composting sites, which are mainly located in periurban areas around the major cities. Their value as an N source will depend primarily on the total N contents and the microbially available N, but their practical use is also influenced by the moisture content (Table 1). An important consideration in the selection of materials is whether they are in high demand for alternative uses, since this value may make them prohibitively expensive for composters. Mushroom composts have been successfully prepared by incorporating blood meal, canola meal, cotton seed meal, guano, malt sprouts (Gerrits 1988; MacCanna 1969; Riethus 1962; Rinker 1991) and brewers’ grains (Beyer and Beelman 1995; Rinker 1991) but these materials also have a fertiliser or animal feed value. In several experiments, excess application of these materials in compost formulations resulted in poorer mushroom yields than moderate applications (Table 2).

**Table 1:** Organic matter nitrogen sources for mushroom compost. Materials currently used or with potential for use in Australasia are in bold text

N source	N	Dry matter	References
	% of DM	%	
Blood, dried meal	12.2	100	(Ministry of agriculture fisheries and food 1976)
Brewers’ grains, dried	3.4	92	(Beyer and Beelman 1995)
Brewers’ grains	3	24	(Noble and Dobrovin-Pennington 2007)
**Canola/rape seed meal**	**3.3**	**85**	**(Stamets** 2000**)**
Cattle slurry	< 2.8–10	10, < 14	(Dawson 1978; Grabbe 1974)
Cocoa meal	4.2	93	(Noble et al. 2002)
**Cottonseed meal**	**6.5**	**92**	**(Stamets** 2000**)**
**Cotton trash**	**1.5**	**91**	**(Stamets** 2000**)**
Digestate, poultry manure	3.5	31.3	Noble (unpublished)
**Feather meal**	**4.9**	**67**	**(Ministry of agriculture fisheries and food** 1976**)**
Fish solubles	5	50	(Schisler and Patton 1974)
**Glasshouse crop haulms**	**1.8**	**11**	**(Noble** 2005**)**
**Grape marc**	**1.8–2**	**27–32**	**(Noble** 2005**;** **Pardo et al.** 1995**;** **Stamets** 2000**)**
Guano	8–15	> 94	(Schnug et al. 2018)
Hop waste, dried	3.3	90	(Noble et al. 2002)
Horn meal	14.5	90	(Ministry of agriculture fisheries and food 1976)
**Horse manure**	**1.3**	**37**	**(Gerrits** 1988**)**
Malt sprouts, dried	4.3	92.6	(Stamets 2000)
**Paunch grass**	**3–3.5**	**15**	**(Environment protection authority** 2017**)**
Pig manure	1.9	23	(van Loon et al. 2004)
**Poultry manure, caged**	**1.5–4.7**	**25–67**	**(Ministry of agriculture fisheries and food** 1976**;** **Wiedemann et al.** 2006**)**
**Poultry manure, broiler**	**4.5–5.4**	**60–66**	**(Gerrits** 1988**;** **Noble and Gaze** 1994**;** **Wiedemann et al.** 2015**)**
**Poultry manure, deep litter**	**2.2–2.7**	**48–79**	**(Ministry of agriculture fisheries and food** 1976**;** **Wiedemann et al.** 2006**)**
Sea algae meal	0.7	32	(Ministry of agriculture fisheries and food 1976)
**Soya bean meal**	**7.1–7.4**	**91**	**(Stamets** 2000**)**
Sugarcane bagasse	0.2	19	(Kneebone and Mason 1972; Stamets 2000)
**Vegetable wastes**	**1.8**	**13**	**(Noble and Dobrovin-Pennington** 2007**)**
**Wool waste**	**14**	**90**	**(Noble and Dobrovin-Pennington** 2007**)**

**Table 2:** Effect of compost organic nitrogen sources on mushroom yield in experiments conducted in medium- and large-scale facilities (> 1 tonne compost)

N source	Inclusion, %*		Yield, % of control		Control compost	Reference
	Min	Max	@ Min	@ Max		
Blood, dried meal	0.45–1.25	1.96–3.21	110–126	77–115	Horse manure	(Riethus 1962)
Blood, dried meal	0.27 N	0.4 N	141	146	Horse manure	(MacCanna 1969)
Brewers’ grains	31.25		126		Horse manure	(Riethus 1962)
Brewers’ grains	9.15		100		Horse manure	(Schisler and Patton 1974)
Canola meal	12 dm		106		Horse manure	(Rinker 1991)
Cattle slurry	10	40	100	83	Horse manure	(Grabbe 1974)
Cattle slurry	35 dm		134		Straw, poultry manure	(Dawson 1978)
Cocoa meal	50 N	100 N	72	40	Straw, poultry manure	(Noble et al. 2002)
Cotton seed meal	2.5		128		Horse manure	(Bech and Riber Rasmussen 1969)
Cotton seed meal	0.14 N	0.20 N	139	123	Horse manure	(MacCanna 1969)
Cotton seed meal	5		109		Horse manure	(Gerrits 1977b)
Digestate, poultry manure	60		88		Straw, poultry manure	Noble (unpublished)
Fish solubles	5	11.8	100	100	Horse manure	(Schisler and Patton 1974)
Grape marc	11		114		Straw, horse and poultry manures	(Pardo et al. 1995)
Guano	1.05–2.09	3.34	72–125	112	Horse manure	(Riethus 1962)
Hop waste, dried	100 N		88		Straw, poultry manure	(Noble et al. 2002)
Horn meal	0.89	1.78	62–118	0–92	Horse manure	(Riethus 1962)
Pig manure	81	96	69	0	Straw, poultry manure	(van Loon et al. 2004)
Poultry manure, broiler	9		89–111		Horse manure	(Gerrits 1977b; Gerrits 1989)
Poultry manure, broiler	33	50	100	103	Straw, poultry manure	(Gerrits 1989)
Poultry manure, broiler	20	62.5	100	146	Straw, poultry manure	(Noble and Gaze 1996)
Sea algae meal	2.49	3.13	90	94	Horse manure	(Riethus 1962)
Sugar cane bagasse	100		100		Horse manure	(Kneebone and Mason 1972)
Vegetable wastes	25		105		Straw, poultry manure	(Noble and Dobrovin-Pennington 2007)
Wool waste	5		92		Straw, poultry manure	(Noble and Dobrovin-Pennington 2007)

* - % w/w of fresh weight, dry matter (dm) or total nitrogen content of N sources (N)

Due to its moderately high N content, widespread availability and low alternative value, poultry manure has been a standard mushroom compost ingredient for many decades (Table 1). Australia produces over 1 million tonnes of poultry litter annually, though the composition of the litter and its suitability for mushroom compost production depends on the type of poultry production and the bedding material (Gerrits 1988; Wiedemann et al. 2015; Wiedemann et al. 2006). Poultry manure with readily degradable bedding material such as straw is more suitable for mushroom composting than manure with sawdust or wood shavings, which can encourage the growth of green moulds (den Ouden 2016). Broiler poultry manure is preferred because of its lower moisture content and easier handling and storage, although there can still be large variability in the quality of poultry manure obtained from apparently similar sources. For example, Cormican and Staunton (1984) recorded a range in N content from < 2.1 to > 3.6% of DM within Irish sources of broiler manure. Manure from egg laying hens is also used in some countries (Cormican and Staunton 1984; Gerrits 1988), particularly where it is first made into a slurry. Ammonia suppressants are applied to the bedding by some poultry farmers to reduce the injurious effects of ammonia on the birds. The presence of these ammonia suppressants does not affect composting or mushroom cropping. For example, use of poultry manure containing a suppressant based on monocalcium phosphate led to only slightly elevated ammonia levels during composting (Beyer et al. 2000), while poultry litter treated with a sodium hydrogen sulphate suppressant did not affect ammonia emissions or compost N during composting (Beyer et al. 2000; Gonzalez-Matute and Rinker 2006). Neither suppressant changed the subsequent mushroom cropping performance.

Where straw is the main C source in the compost, there is an optimum inclusion rate of poultry manure, depending on the C and N analysis (Gerrits 1988; Noble and Gaze 1996). Where horse manure is the main C source in the compost, addition of excess poultry manure can readily lead to an over-supply of N and reduced mushroom yield (Gerrits 1989). However, researchers from the 1960s onwards have found that mushroom yields from horse manure composts were improved by the addition of a range of organic N sources, including poultry manure, providing that this did not result in residual ammonia in the compost (Table 2). Examples include the following. Ross (1969) obtained mushroom yields comparable with those from horse manure composts using composts prepared from strawy bullock manure or pig slurry and straw. Grabbe (1974) replaced water with liquid cattle slurry in a horse manure-based Phase I compost and obtained the same mushroom yield. Dawson (1978) obtained mushroom yields at least comparable with straw and poultry manure compost when 70% of the poultry manure was replaced by an equivalent amount of N as cattle manure. Sugarcane bagasse and straw have been used to produce composts with comparable mushroom yields to those obtained from horse manure composts (Kneebone and Mason 1972; Peerally 1981). Digestate fibre from the anaerobic digestion of poultry manure (Table 2), food or crop wastes has been used in the production of mushroom substrates (Noble et al. 2002; Stoknes et al. 2008).

During composting with wheat straw, the use of vegetable wastes, dried hop waste and brewers’ grains released less ammonia than poultry manure during composting but produced similar mushroom yields (Noble et al. 2002; Noble et al. 2006). Similarly, crop haulm and residues from glasshouse crops such as peppers and tomatoes contain moderate amounts of available N and could be used in mushroom compost formulations although their availability is seasonal (Noble 2005). Other organic materials (chipboard waste, cocoa meal and shells, wool waste and dried digestate fibre) which had total N contents above 2% of DM, only released small amounts of ammonia during composting and resulted in poor mushroom yields (Table 2). However, these materials may be suitable with longer composting periods to enable the release of N. Due to its current low price, low quality wool is now a significant by-product of the sheep meat industry, and it is used in the production of horticultural composts (Williams 2020). Paunch grass, the undigested contents of animal carcasses, is a by-product from abattoirs, has moderate N content but it is high in moisture (Table 1). It could be used in mushroom composting where high Phase I bunker temperatures would meet the regulatory requirements for animal waste disposal (Environment protection authority 2017).

Recycling of spent mushroom compost and green wastes into Phase I ingredients was examined by Noble et al. (2006) and reviewed recently by Zied et al. (2020). Use of these materials can make a substantial contribution to the circular economy, and an up-to-date inventory of the types, quantities and supply of by-products and wastes from agricultural and food production industries is urgently needed. Such a review should take into account recent changes in these sectors, paying particular attention to whether materials are suitable for both conventional and organically approved mushroom production.

## Inorganic N sources

Various chemical fertiliser or inorganic N sources have been used in mushroom compost formulations, including urea, ammonium sulphate and nitrate, calcium ammonium nitrate, calcium nitrate and cyanamide (Table 3). Mushroom yields increased after the addition of ammonium sulphate to Phase I compost, providing that this was accompanied by an addition of calcium carbonate (MacCanna 1969; Riber Rasmussen 1965). When a proportion of the poultry manure in compost was replaced with ammonium sulphate (without calcium carbonate) compost pH was slightly reduced, but there was no effect on mushroom yield (Gerrits 1977a). Ammonium sulphate is widely available since it is a by-product of sulphuric acid scrubbing of composting emissions before biofiltration, and it is therefore a cheap source of N. If combined with calcium carbonate, it also obviates the need for gypsum in mushroom compost (Gerrits 1988; Riber Rasmussen 1965) (see below).

**Table 3:** Effect of compost inorganic nitrogen sources on mushroom yield in experiments conducted in medium- and large-scale facilities (> 1 tonne compost)

N source	N	Inclusion, %*		Yield, % of control		Control compost	Reference
	% w/w	Min	Max	@ Min	@ Max		
Ammonium nitrate	35	0.37	0.72	99–116	113–129	Horse manure	(Riethus 1962)
Ammonium sulphate	21.2	0.12		136		Horse and poultry manures	(Riber Rasmussen 1965)
Ammonium sulphate		0.27 N	0.40 N	134	117	Horse manure	(MacCanna 1969)
Ammonium sulphate		0.4		100–105		Horse and poultry manures	(Gerrits 1977a)
Ca ammonium nitrate	27	0.7		108		Horse manure	(Gerrits 1977b)
Calcium nitrate	17	0.27 N	0.40 N	107	86	Horse manure	(MacCanna 1969)
Urea	46.7	0.17–0.66	0.83	58–112	12	Horse manure	(Riethus 1962)
Urea		0.57		75		Horse manure	(Bech and Riber Rasmussen 1969)
Urea		0.35		100–105		Horse manure	(Gerrits 1977b)
Urea		50 N		79		Straw, poultry manure	(Noble et al. 2002)
Urea formaldehyde	38.9	0.68		49		Horse manure	(Bech and Riber Rasmussen 1969)
Urea formaldehyde		0.27 N	0.40 N	107	105	Horse manure	(MacCanna 1969)

* - % w/w of fresh weight or total nitrogen content of N sources (N)

Urea is a more readily available form of N to compost microbes than ammonium sulphate and results in a more rapid release of ammonia from compost (Noble et al. 2002). It can be added during the pre-wetting of raw materials where it is less likely to cause odour nuisance than poultry manure (Noble et al. 2002). However, composts prepared with urea rather than with ammonium sulphate led to reduced mushroom yields (Bech and Riber Rasmussen 1969). Pardo et al. (1995) added a combination of urea (8.3 kg), ammonium sulphate (4.2 kg) and gypsum (38.9 kg) per tonne of straw and manure; replacing poultry manure with an equivalent amount of N as urea resulted in higher N losses during composting with wheat straw; conversely, replacement with ammonium sulphate led to lower losses (Noble et al. 2002). Composts in which either of these two inorganic N sources replaced 50–100% of the poultry manure-N produced lower mushroom yields than when poultry manure provided the sole N source (Noble et al. 2002). This has also been found more generally, with excess application of inorganic N in compost formulations usually producing poorer mushroom yields than application of moderate amounts (Table 3).

## Recycled water

Recycled compost leachate (so-called goody water) can be a significant source of compost N if it makes up a high proportion of the water added during pre-wetting of the raw materials. The composition of goody water is influenced not only by the compost ingredients but also by the wetting and composting procedures and rainfall on outdoor compost yards. Goody water samples collected from the storage tanks or pits of 14 mushroom composting sites in Britain and Ireland contained between 3.2 and 6.4 mg N L^−1^, mainly in the form of urea, ammonium N, P-serine and other amino acids (Noble et al. 2006). Analysis of goody water from an Australian composting site showed much higher levels of 4–11 g L^−1^ of total N, measured over a period of one month (Safianowicz et al. 2018).

## Influence of gypsum on compost nitrogen

Gypsum was originally added to mushroom compost to improve the physical structure by flocculating colloids and preventing greasiness and anaerobic conditions, but this function has been made unnecessary by the use of shorter and more aerated composting systems (Fermor et al. 1985). However, better mushroom yields were still obtained with compost where gypsum was added at 25 kg per tonne than when gypsum was omitted (Gerrits 1977a), which the authors attributed to the effect of gypsum in reducing compost pH and decreasing the dissociation of ammonium N into ammonia. Addition of gypsum (25 kg t^−1^) to a straw and poultry manure compost led to normal mushroom yields of 300 kg t^−1^ pasteurised compost, whereas no mushrooms grew on the same compost without the addition of gypsum (Noble, unpublished). A further study (Riber Rasmussen 1965) found no effect of adding gypsum to compost, though the formulation used included both ammonium sulphate and calcium carbonate, which would react to form gypsum. Removing gypsum from the compost formulation altogether increased the rate of cellulase and xylan degradation, so that the time required to achieve a defined compost quality was reduced by approximately 20% (Mouthier et al. 2017). Increasing the rate of gypsum inclusion from 28 to 84 kg t^−1^ compost did not affect compost pH or mushroom yield (Beyer and Beelman 1995).

The work described above indicates that the beneficial effect of gypsum on mushroom compost is most likely due to the sulphate ions reacting with ammonia to form ammonium sulphate, thereby stabilising compost ammonium N. This effect is partially counteracted by the calcium ion content of gypsum, which would tend to increase compost pH and destabilise the ammonium-N in the compost. Mushrooms do not have a significant calcium requirement (Gerrits 1988) and there is an abundance of calcium from the lime content of the casing material which is used to cover the compost to induce sporophore production. It may therefore be more effective to add dilute sulphuric acid to compost to stabilise the ammonium N, a technique which is used to remove ammonia from composting emissions before biofiltration. The cost of sulphate ions in sulphuric acid is significantly less than in gypsum, although the cost and safety of spray application of acid would also need to be considered.

## Compost supplements

The addition of protein-containing supplements to mushroom-colonised or ‘spawn-run’ (Phase 3) compost to increase mushroom yields and quality is now practised on most mushroom farms and has a considerable effect on yield and quality (Carrasco et al. 2018). The benefits are greater than with adding supplements to pasteurised (Phase 2) compost at spawning, where there is more competition for nutrients from other micro-organisms. In terms of mushroom yield, the benefit of adding supplements increases with ‘meagre’ composts that have low N content, although yields are still increased by supplementation of composts made with an ‘adequate’ N supply (Gerrits 1988; Noble and Gaze 1994). This indicates that the availability and/or type of protein in such composts is still sub-optimal and restricted by the amount of ammonium-N which can be present in the compost formulation. A wide range of materials of plant and animal origin have been tested for use as supplements. Seed meals and processed products, particularly from cottonseed and soya bean, generally give the best results, with performance related to crude protein content (Gerrits 1988; Randle 1985). The substances are usually treated with formaldehyde and/or coated to reduce the immediate availability of protein, in order to prevent a surge in compost temperatures, and also reduce the uptake of nutrients by competitor moulds. Commercial supplements are based on formaldehyde-treated soya bean meal and other biological by-products and are added to Phase 3 compost at 0.5 to 1.6% w/w, with expected mushroom yield increases of 10–30% (Burton and Noble 2015; den Ouden 2016; Gerrits 1988). Randle and Smith (1986) have calculated that a mushroom yield increase of at least 10%, without a change in quality, was required to justify the cost of compost supplementation using such materials, while a more recent estimate by Burton and Noble (2015) has put the typical gross value of the additional mushrooms harvested at six times the cost of the supplement, though this did not include the costs of applying the supplement or of harvesting and marketing the extra mushrooms.

## Compost N sources in Australasia

Phase I mushroom compost is produced on around 12 composting yards across Australia and 4 in New Zealand, each site producing between 60 and 1800 tonnes each week. The composts are based on wheat straw as the main C source (Table 4), unlike many Phase I composts in Europe which are partially or entirely based on horse manure and may include other types of straw such as barley, rye and oilseed rape. Phase I compost N contents are typically 1.8 to 2.2% w/w of DM (Table 4), which is predominantly supplied by poultry manure. Due to a decline in the rice crop in Australia, broiler poultry bedding material based on degradable rice husks is being replaced by wood shavings, leading to more composting yards using layer hen manure (Wiedemann et al. 2006) and increasing the need for alternative N sources. Some composting yards substitute some of the N supplied by the poultry manure with other materials, adding up to 6% inorganic N or 20% organic N replacements. To wet the composts, all the composting yards supplement fresh water with at least 50% recycled compost leachate. This addition of recycled compost leachate would theoretically account for less than 5% of the N added to the compost formulations, calculated from the average tonnages of Phase I compost produced, the moisture and N contents of the composts (Table 4), typical moisture and N contents of the raw materials (Table 1), and a DM loss of 30% during Phase I composting (den Ouden 2016; Gerrits 1988). However, this figure still needs to be verified with actual process measurements of e.g. applied water volumes, DM losses, N losses due to leaching and ammonification during Phase I and recycled water N content.

**Table 4:** Current formulations used on some Australasian mushroom composting yards and typical Phase I compost analyses. All sites use wheat straw at 54–61% w/w of the raw materials and add gypsum at 25–30 kg/t compost

Parameter	Compost yard					
	A	B	C	D	E	F
Horse manure	0	0	✓	0	0	0
Poultry manure, laying	✓	✓	✓	✓	0	0
Poultry manure, broiler	0	✓	✓	0	✓	✓
Feather meal	0	0	✓	0	0	0
Canola meal	0	✓	0	0	0	✓
Cotton trash	0	✓	0	0	0	0
Cottonseed meal	0	0	✓	0	0	0
Soya bean meal	✓	0	0	0	0	0
Urea	✓	0	✓	0	✓	0
Ammonium sulphate	0	0	0	✓	0	0
Recycled water, %	50	✓	100	50	✓	90
Dry matter, % w/w	27.0	26.0	24.6	27.0	25.0	26.2
N (g kg^−1^dry matter)	17.0	18.5	22.4	18.0	22.0	17.6
NH_4_^+^ (g kg^−1^dry matter)	n.d.	4.0	4.0	2.0	4.5	1.8
Ash (g kg^−1^dry matter)	272.4	n.d.	193.9	225.0	n.d.	103.0
pH	8.05	n.d.	8.26	8.25	8.3	8.1

*n.d.* not determined

A range of organic matter N sources is available for mushroom composting in Australasia, but not all are equally suitable for widespread use, and their uptake by mushroom composters will depend on a number of factors. The most important of these are their content of microbially available N on a weight and bulk volume basis, and how successful similar materials have been in mushroom cultivation tests in other countries. However, more practical details are also critical, such as their year-round availability and/or tolerance to storage (and requirements for bulk storage), their uniformity and the absence of physical and chemical contaminants. Importantly, commercial viability will also depend on such N sources being widely available and not having significant alternative competitor value for applications such as such as animal feed, fertiliser or biofuels.

Organic N sources which are currently used to replace broiler poultry manure in Australasia are highlighted in Table 1. Materials with high moisture contents such as crop haulms, vegetable wastes, grape marc and paunch grass would only be viable if the sources are close to the composting yards and would require readjustment of the water applications made to the compost. Wool wastes could be used but would need longer composting processes to enable the N content to become available. The currently most used N source, poultry manure, even if used at an optimum inclusion rate, does not obviate the need for protein supplements in the prepared substrate to increase mushroom yields. Around 3000 tonnes of soya protein-based compost supplements are imported by the Australasian mushroom industry annually. This may offer an opportunity to replace these imports with locally produced supplements based on alternatives to soya, depending on the availability and suitability of by-product protein sources.

## Conclusion and outlook

Commercial edible mushrooms are cultivated almost entirely on substrates derived from agricultural waste streams, particularly cereal straw and poultry manure, and their production is therefore rightly viewed as part of the circular economy. In principle, a wide range of alternative agricultural wastes could be used, but the choice is rigidly controlled by the need to achieve the correct balance of available C and N sources in the mix. This balance maximises composting efficiency and balances the rate of ammonia production during composting with the accumulation of compost microbial biomass and with the final mushroom yield. However, changes in broiler chicken husbandry have led to a reduction in the N content of available manure, so alternative N sources are urgently needed. A range of different N sources has already been identified that enable the compost protein content to be increased without forming damaging levels of ammonia, and their use could potentially be improved by the application of sulphuric acid to replace gypsum. Further research is required to optimise the timing of N addition, so that this releases ideal amounts of ammonia for straw softening while retaining sufficient N to support growth and development of the microbial community needed both for lignocellulose breakdown and for subsequent growth of the mushroom mycelium. This research will build on recent advances in our understanding of the microbial dynamics of mushroom composting and allow us to develop processes that optimize feedstock conversion into compost of reproducibly high quality and support the development of microbial communities that maximise mushroom yield.

## Data Availability

All data supporting the findings of this review article are available within the paper or in the cited sources.
